# Simultaneous detection of *BRCA* mutations and large genomic rearrangements in germline DNA and FFPE tumor samples

**DOI:** 10.18632/oncotarget.11259

**Published:** 2016-08-12

**Authors:** Márton Zsolt Enyedi, Gábor Jaksa, Lajos Pintér, Farkas Sükösd, Zoltán Gyuris, Adrienn Hajdu, Erika Határvölgyi, Katalin Priskin, Lajos Haracska

**Affiliations:** ^1^ Institute of Genetics, Biological Research Centre of the Hungarian Academy of Sciences, Szeged 6726, Hungary; ^2^ Delta Bio 2000 Ltd., Szeged 6726, Hungary; ^3^ Department of Pathology, Faculty of Medicine, University of Szeged, Szeged 6720, Hungary

**Keywords:** NGS, BRCA1-BRCA2, germline, multiplex PCR, FFPE

## Abstract

The development of breast and ovarian cancer is strongly connected to the inactivation of the *BRCA1* and *BRCA2* genes by different germline and somatic alterations, and their diagnosis has great significance in targeted tumor therapy, since recently approved PARP inhibitors show high efficiency in the treatment of *BRCA*-deficient tumors. This raises the need for new diagnostic methods that are capable of performing an integrative mutation analysis of the *BRCA* genes not only from germline DNA but also from formalin-fixed and paraffin-embedded (FFPE) tumor samples. Here we describe the development of such a methodology based on next-generation sequencing and a new bioinformatics software for data analysis. The diagnostic method was initially developed on an Illumina MiSeq NGS platform using germline-mutated stem cell lines and then adapted for the Ion Torrent PGM NGS platform as well. We also investigated the usability of NGS coverage data for the detection of copy number variations and exon deletions as a replacement of the conventional MLPA technique. Finally, we tested the developed workflow on FFPE samples from breast and ovarian cancer patients. Our method meets the sensitivity and specificity requirements for the genetic diagnosis of breast and ovarian cancers both from germline and FFPE samples.

## INTRODUCTION

Next-generation sequencing (NGS) technologies have reshaped the image of molecular biology. These rapidly evolving technologies provide unprecedented scale and efficiency in DNA sequencing [1] with many potential advantages over traditional Sanger sequencing [2]. The introduction of benchtop NGS platforms opened the way for clinical diagnostic laboratories to incorporate this technology into their daily diagnostic routine. Performance comparison of different benchtop NGS platforms showed substantial differences between them in the quality of data and throughput capacity [3]. These differences have to be considered carefully when searching for the most suitable NGS platform to achieve specific diagnostic requirements and goals. Parallel to the spreading of different NGS methods, several target-enrichment technologies have also been introduced to isolate and prepare regions of the genome for NGS [4]. However, the efficiency and specificity limitations of different target-enrichment methods still represent a bottleneck for targeted NGS [5]. The diversity of sample preparation and NGS techniques used in research can offer a wide spectrum of solutions for DNA sequencing in the clinical practice. However, performance and quality criteria expected from NGS are markedly different in diagnostics and research. To meet regulatory requirements, clinical sequencing assays must be robust and reproducible, with well-defined sensitivity and specificity values [6] so that results can be applied with confidence when making treatment decisions [7].

Germline mutations of *BRCA1* and *BRCA2* predispose to hereditary breast and ovarian cancer syndrome (HBOCS) representing up to 10% of all breast cancers diagnosed annually [8]. Pathogenic mutations in these genes confer an estimated 40% to 85% lifetime risk of breast cancer and a 15% to 40% lifetime risk of ovarian cancer [9, 10]. Selection of women for genetic testing of *BRCA1* and *BRCA2* follows general guidelines based on their family history of cancer [11]. However, not all HBOCS patients fulfil these criteria mostly because of paternal inheritance of the susceptibility or the effect of a small family. The mutational status of these patients often remains unclarified [12, 13].

*BRCA* genes are also involved in the development of sporadic breast and ovarian tumors. Several studies have reported somatic *BRCA1* and *BRCA2* mutations in a considerable proportion of breast and ovarian cancers [14, 15]. The mutational status of the *BRCA* genes is important for selecting patients for personalized treatment, as patients carrying a germline or somatic *BRCA* mutation have shown to give a positive response to poly(ADP-ribose) polymerase-inhibitors (PARPi) [16, 17]. Recently, several studies have pointed out that the miRNA expression pattern also has a significant effect on the progress and outcome of breast and ovarian cancer [18, 19]. Assessing the *molecular signature* of tumor samples is thus a crucial step in the proper management of breast and ovarian cancer patients.

In clinical laboratories, the diagnostic sequencing of *BRCA1* and *BRCA2* is often performed by Sanger sequencing of individually amplified PCR products [20, 21]. This method is suitable for the detection of single-base substitutions, small insertions, and deletions. For the detection of copy number variations (CNV), alternative methods such as multiplex ligation-dependent probe amplification (MLPA) have to be used [22]. Due to the lack of mutational hotspots in the *BRCA* genes and their relatively large size, the traditional capillary sequencing-based diagnostic process combined with MLPA analysis represents an expensive and time-consuming solution. In addition, tumor samples are usually heterogeneous containing normal and tumor cells in variable amounts, which often makes Sanger sequencing and MLPA analysis unreliable. The advent of different NGS and target-enrichment methods offered the possibility to relocate the *BRCA1* and *BRCA2* mutation detection workflow onto these high-throughput platforms. Several NGS systems have already been evaluated using these two genes. The majority of the studies reporting *BRCA* diagnostic methods focus on the detection of germline mutations using blood samples, from which high-quality genomic DNA can be prepared [13,23–27]. However, the majority of tumor samples are formalin-fixed paraffin-embedded (FFPE) clinical specimens and thus DNA isolated from these samples has specific characteristics which often make the subsequent mutational analysis difficult: the amount of DNA isolated from FFPE samples is often limited, and the quality is poor due to deamination and cross-linking during formalin-fixation. Only a limited fraction of the available studies present some solution to somatic *BRCA* mutation detection from FFPE samples [28, 29].

Given the diversity of sample types (blood, FFPE) and possible mutations (SNP, indels, CNVs) in *BRCA* analysis and due to the appearance of PARP inhibitors, there is a strong clinical demand for an integrative diagnostic solution to detect the various mechanisms of *BRCA* inactivation.

In this work, we present a multi-sided *BRCA* diagnostic method based on multiplex PCR amplification, next-generation sequencing, and computational variant identification that is versatile to face all the challenges mentioned above. Using previously validated sample pools, we optimized and validated the sequencing and mutation detection performance on the two most popular benchtop sequencing platforms: Illumina MiSeq and Ion Torrent PGM. We further demonstrate that the sample processing method used for the detection of germline mutations is also suitable for the identification of whole exon deletions and duplications. Most importantly, we show that the method performs well on FFPE samples of breast and ovarian tumors and is suitable for somatic mutation detection. Therefore, combining the multiplex PCR/NGS sequencing method with the appropriate NGS platform results in a complete integrative and robust diagnostic pipeline for *BRCA1* and *BRCA2* analysis.

## RESULTS

**Figure 1:**
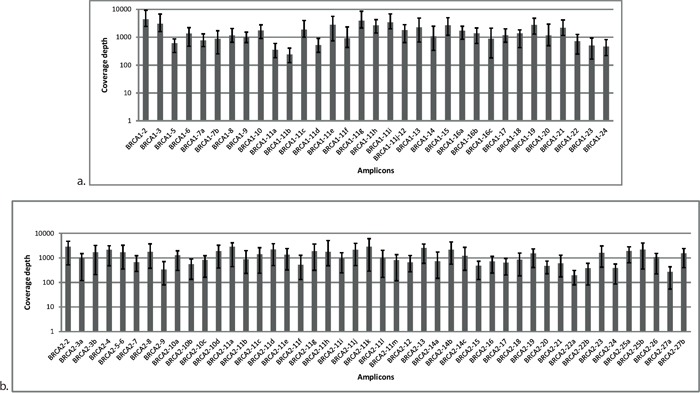
Distribution of coverage for each amplicon in *BRCA1* a. and *BRCA2* b. originating from Illumina MiSeq sequencing data Each amplicon was surveyed at multiple reference points. Average was calculated from 24 samples. Error bars represent the minimum and maximum values of coverage in the respective amplicon.

A total of 24 DNA samples with known pathogenic germline mutations were used to calibrate the routine procedure, that is, multiplex PCR amplification, library preparation, and bioinformatics analysis parameters. To adequately address diagnostic issues, this sample set was composed of difficult cases, for example, insertions and/or deletions of various sizes and mutations occurring in homopolymer regions of *BRCA1* and *BRCA2* (Table 1). Samples were pooled and sequenced together but remained readily identifiable using barcodes (indices) ligated to the amplicons during library preparation. The application of optimized experimental conditions resulted in a full coverage of the *BRCA1* and *BRCA2* regions of interest with uniform representation of each PCR amplicon in the coverage distribution (Figure 1). The average number of reads mapped per sample was 133,067 resulting in an average coverage of 689x.

**Table 1:** *BRCA1* and *BRCA2* variants in the 24 Coriell Cell Line Reference Samples used as training set for the optimization of the workflow

Nr	DNA ID	Gene	Mutation	Exon
**1**	NA14090	*BRCA1*	c.66_67delAG	3
**2**	NA14638		c.213-11T>G	5
**3**	NA14684		c.797_798delTT	11
**4**	NA14094		c.1175_1214del40	11
**5**	NA14093		c.1204delG	11
**6**	NA13709		c.2068delA	11
**7**	NA13712		c.2155_2156insA	11
**8**	GM14096		c.3481_3491delGAAGATACTAG	11
**9**	NA13705		c.3756_3759delGTCT	11
**10**	NA14634		c.4065_4068delTCAA	11
**11**	NA13710		c.4327C>G	13
**12**	NA14637		c.4327C>T	13
**13**	NA13708		c.4752C>G	16
**14**	NA14095		c.5200delG	18
**15**	NA14092		c.5201T>C	18
**16**	NA13715		c.5326_5327insC	20
**17**	NA13714		c.5319_5320insC	21
**18**	NA14636		c.5621_5622insA	24
**19**	NA14623	*BRCA2*	c.125A>G	3
**20**	NA14624		c.5718_5719delCT	11
**21**	NA14170		c.5946delT	11
**22**	NA14639		c.6198_6199delTT	11
**23**	NA14622		c.6275_6276delTT	11
**24**	NA14626		c.9976A>T	27

During the optimization of the mutation detection algorithm, we paid special attention to large indels represented by two samples in the training set: an 11-bp deletion (GM14096) and a 40-bp-long deletion (NA14094). These indels were correctly mapped and identified by the in-house developed NGSeXplorer software (Supplementary Figure S1). As in the validation set we did not have the possibility to include other samples with long indels, we evaluated the reproducibility of the identification of large indels by analyzing the above mentioned two samples in two independent sequencing runs. The coverage data and variant calling results of this reproducibility test are summarized in Supplementary Table S1.

**Figure 2:**
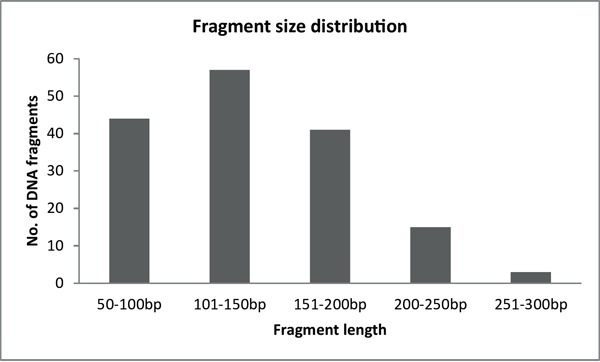
Fragment size distribution of *BRCA1-2* samples after the enzymatic fragmentation of multiplex PCR products

After the initial optimization process, we blindly validated the diagnostic method on 20 samples each containing a unique pathogenic mutation (Table 2). At the same time, we compared the mutation detection performance of the two most popular NGS platforms: Illumina MiSeq and Ion Torrent PGM. Sequencing parameters, number of reads, and coverage values were evaluated for both sequencing platforms and are summarized in Table 3. The average read length was similar for both sequencing platforms and was mainly determined by the length of the DNA fragments generated after the restriction enzyme digestion of PCR products (Figure 2). Each manufacturer uses unique software implementations to generate base-quality score predictions, thus, direct comparison of these scores between different platforms is difficult, even taking into account that Ion Torrent PGM generally underestimates quality scores while Illumina MiSeq slightly overestimates them [3]. Based on the alignment of sequencing reads, we conclude that the substitution error rates produced by the two platforms are similar (0.29 and 0.23 substitutions per 100 bases for Illumina and Ion Torrent, respectively). Comparison of the frequency of insertions and deletions demonstrated that the Ion Torrent PGM reads had 1.27 insertions and 0.67 deletions per 100 bases (Table 3), while in the case of Illumina MiSeq insertion and deletion events were nearly absent (0.01 and 0.04, respectively). The allelic ratio for mutation detection was adjusted to 25% both for single nucleotide variation (SNV) and indel detection, with a minimum depth of coverage of 50x.

**Table 2:** List of *BRCA* mutant samples included in the validation set

Gene	Mutation type	Description
***BRCA1***	SNVs (polymorphisms	c.181T>G
	excluded)	c.5251C>T
		c.5074 G>C
	Insertions/deletions	c.843_846delCTCA
		c1016_1017insA
		c.1961delA
		c.2985delTCTCA
		c.3700_3704delGTAAA
		c.3756_3759delGTCT
		c.4065_4068delTCAA
		c.5266dupC
	Large rearrangements*	del(ex21_22)
		dup(ex13)
***BRCA2***	SNVs (polymorphisms	c.5645C>A
	excluded)	
	Insertions/deletions	c.476-9_476-8insT
		c.1813dupA
		c.5073dupA
		c.5351_5352insA
		c.5946delT
		c.7910-7914delCCTTT
		c.9098_9099insA
		c.9403delC

* The two large rearrangements in the case of *BRCA1* are also included in this table.

**Table 3:** Summary of read number, coverage, and mapping results of the two NGS platforms

Characteristics	Illumina MiSeq	Ion torrent PGM
**Flow cell/chip type**	Standard	316
**Total no. of reads**	14M	915k and 1.25M
**Mean read length**	99.3bp	99.24bp
**Average quality of reads (Phred)**	33.79	24.24
**Average no. of reads/sample**	247,749	99,774
**%mapped reads**	76%	88%
**Coverage, mean [min, max]**	1,262 [171, 3642]	439 [75, 1326]
**% of target area with ≥ 50 fold coverage**	99.93	99.68
**% of insertions**	0.01	1.27
**% of deletions**	0.04	0.67
**% of mismatches**	0.29	0.23

All values are average calculations from the 20 analyzed samples.

### Illumina MiSeq mutation detection

Our in-house developed software (NGSeXplorer) correctly identified the known variants in all 20 retrospective samples and correctly assigned homozygosity and heterozygosity, with no false-positive or false-negative variant predictions (Table 4). Illumina sequencing resulted in an experimental sensitivity [TP/(TP + FN)] of 100% where TP is true-positive and FN is false-negative. In the absence of true-negative variants, specificity was estimated by using the false-positive (FP) rate FP/(FP + TP), resulting in 0%. Sensitivity and specificity values were calculated for the validation set of 20 samples used in the performance comparison between the two platforms.

**Table 4:** Variant calling results from the validation set

Filtered variants		NGS Platform	
		Illumina MiSeq	Ion torrent PGM
**Total**	False-positive	0	26
	False-negative	0	16
	True-positive	140	124
	True-negative		982
**Pathogenic**	False-positive	0	16
	False-negative	0	1
	True-positive	20	19
	True-negative		680

### Ion Torrent PGM mutation detection

During the initial phase of mutation detection, all called variants (1148 variants per 20 samples) were retrieved from the Ion Torrent PGM sequence data set without applying any of the specific bioinformatic filters. After this, different filter thresholds were computed in order to minimize the number of false-positive calls without the introduction of false-negative results (Table 5). The filter thresholds were set as follows: depth test 5, insertion overrun 20%, deletion displacement 30%, HP percentage 25%, and strand bias 35%. Modification of filter thresholds in order to reduce the number of false-positives would lead to the appearance of false-negative results.

**Table 5:** Cumulative application of filters for Ion Torrent PGM data

Filters	Number of variants	
	Total	Pathogenic
**Total variants**	1,148	715
**Depth test**	1,072	669
**Insertion overrun**	565	293
**Deletion displacement**	261	101
**HP percentage**	228	71
**Strand bias**	166	36

From the 20 samples sequenced with Ion Torrent, in the case of 9 samples only the true pathogenic mutations remained after variant filtering. In the case of 10 other samples, 1-4 false-positive pathogenic mutations remained in addition to the real one. In the case of the remaining one sample, the true pathogenic mutation was discarded by one of the variant filtering steps. This was a Cytosine to Adenine substitution (c.5645C>A) followed by a four-mer Adenine repeat. The mutation was filtered out by the *Strand bias* test because the average quality value of the surrounding bases (due to the homopolymer sequence) on the reverse strand was below threshold (Q10), hence, the majority of these reads were ignored during variant detection. In conclusion, Ion Torrent variant calling of pathogenic mutations resulted in an experimental sensitivity of 95.0% and a specificity of 97.3% calculated as follows: [TN/(TN + FP)]. The overall accuracy [TP + TN/(TP + TN + FP + FN)] of the assay was 97.2%.

### Copy number variation detection

**Figure 3:**
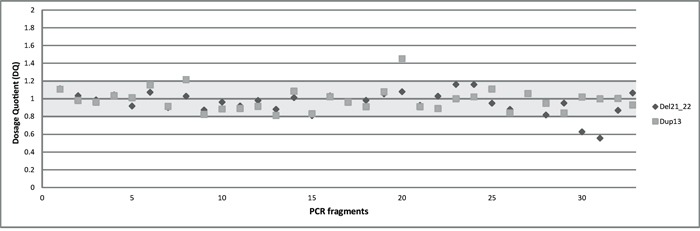
The overall dosage plot for LGR analysis Every dot is the average of the DQ of the amplicons of *BRCA1* in two samples (diamond and square shaped dots).

To identify CNVs in the *BRCA1* gene with our multiplex PCR/NGS sequencing method, we used Illumina MiSeq read counts as readout. We calculated the average DQs for all the 36 *BRCA1* amplicons in four different groups representing the four multiplex reactions used for *BRCA1* amplification. This DQ results from the comparison of normalized amplicon amounts between a test individual and control individuals as detailed in *Materials and Methods*. We presented the four DQs in a single scatter plot. The DQ values of the 36 *BRCA1* amplicons can be separated into three ranges of 0.5 (range 0.55-0.63), 1.0 (range 0.81-1.2), and 1.5 (1.45), corresponding to the number of copies present in the individuals. Based on this segregation, we conclude that in one of the test samples, a deletion of exon 21 and 22 has occurred, while in the other sample a duplication of exon 13 can be observed (Figure 3). The results are in concordance with those obtained with MLPA analysis.

### FFPE sample analysis

The complementation of multiplex amplification with singleplex PCR reactions in case of FFPE samples ensured 100% coverage similarly to the germline samples. The mean depth of coverage was 2009x and 841x for the FFPE/matched normal samples with an average read number of 458,832 and 230,779, respectively. During the variant calling process, we identified pathogenic mutations in four of the ten tumor samples, all of them present in *BRCA1*: a frame-shift insertion (c.5263_5264insC) and two nonsense mutation (c.628C>T and c.5251C>T). Mutational analysis of the matching normal samples revealed that three of the four identified mutations were germline, and only one tumor sample contained a somatic mutation (Table 6) that has not been recorded yet in the COSMIC database (c.628C>T; p. Q210*). All four pathogenic mutations were verified by Sanger sequencing. The newly identified somatic mutation (Q210*) was reported to COSMIC database (accession number: 41127). The tumor samples and their normal match also contained some common germline variants that are present in the majority of the population (Supplementary Table S2). In the case of the mutant and non-mutant FFPE DNA mixture, the mutant allele was detected in the first two admixtures (1/2 and 1/4) by the automated variant calling process. Visual inspection of filtered variants revealed the presence of the mutant allele also in the other two admixtures with frequencies below the variant detection threshold of 10% (Supplementary Table S3). The differences between the expected and observed allele frequencies are possibly due to the difference in the amount of amplifiable DNA in the FFPE samples used to generate the admixtures.

**Table 6:** List of the FFPE and matching normal samples of the ten ovarian cancer patients included in this test

Case nr.	Tissue type	Mutational status
**1**	Normal FFPE	WT
	Tumor FFPE-50%	WT
**2**	Periph. blood	WT
	Tumor FFPE-30%	WT
**3**	Periph. blood	WT
	Tumor FFPE-60%	WT
**4**	Periph. blood	WT
	Tumor FFPE-80%	WT
**5**	Periph. blood	WT
	Tumor FFPE-60%	WT
**6**	Periph. blood	WT
	Tumor FFPE-60%	WT
**7**	Periph. blood	WT
	Tumor FFPE-80%	*BRCA1*: p.Q210*
**8**	Normal FFPE	*BRCA1*: p.Ser1755?fs
	Tumor FFPE-80%	*BRCA1*: p.Ser1755?fs
**9**	Periph. blood	*BRCA1*: p.Ser1755?fs
	Tumor FFPE-30%	*BRCA1*: p.Ser1755?fs
**10**	Periph. blood	*BRCA1*: p.R1751*
	Tumor FFPE-70%	*BRCA1*: p.R1751*

Tumor cell percentage in FFPE samples was estimated visually by molecular pathologist. Lymphocyte DNA isolated from peripheral blood was originally used as a default reference normal DNA, however, in some cases this was not possible and thus was replaced by FFPE DNA from non-tumorous regions of the tissue-slide. Mutational status of the samples determined by NGS is also illustrated. Detailed list of mutations and neutral variants found in these 10 sample-pairs can be found in Supplementary Table S2.

## DISCUSSION

In this paper, we describe an integrative diagnostic solution for the analysis of the *BRCA1* and *BRCA2* genes. This method offers the possibility of detecting different mutation types such as point mutations, small deletions, and exon or allele losses both from germline and FFPE samples. The complete workflow is based on a multiplex PCR amplification and enzymatic fragmentation strategy to generate patient DNA library followed by NGS sequencing and data analysis. The use of restriction enzymes for NGS fragment library preparation is not a widespread method, but it has significant advantages when the copy number of the DNA to be sequenced is limited such as often in the case of FFPE-derived samples. For example, MseI restriction digestion has been successfully used for DNA fragmentation in single-cell whole-genome analyzing studies [30, 31]. Here we complemented the MseI-mediated fragmentation with two other restriction enzymes that recognize four-base motifs: Csp6I and FspBI. All three restriction enzymes generate TA overhangs at the 5′end of DNA fragments, which makes the subsequent ligation of adapter sequences easier.

For the optimization of the methodology, we used a training set of 24 DNA samples from stem cell lines with previously identified germline *BRCA* mutations. After adequate optimization of the multiplex PCR conditions and the library preparation process, all 24 samples were sequenced on an Illumina MiSeq platform. For NGS data analysis, initially we used platform-specific bioinformatics tools (Torrent Suite Software in the case of Ion Torrent and MiSeq Reporter software for Illumina). However, when it became clear that these tools do not fulfil all our expectations we developed our own bioinformatics pipeline, the NGSeXplorer. The software contains an accurate alignment module, a coverage generator, and a variant calling unit with several variant filtering algorithms for Ion Torrent PGM sequencing data. The software uses the Breast Cancer Information Core [32], the UMD, and the Ensembl.org databases for variant annotation. The most important factor for accurate mutation detection during NGS is the complete coverage of the target region as well as the optimal depth of coverage inside the target region. Coverage results of the training sample set clearly demonstrated that 100% of the target region was successfully covered by the PCR fragments amplified in multiplex setting. A major challenge in the multiplex PCR enrichment method is the uniform representation of different amplicons in the reactions and later in the coverage data. This was successfully overcome here by adjusting the primer concentrations and PCR conditions (Figure 1). Regarding the minimal depth of coverage for germline mutation detection, we took into account the range of 50-fold coverage, as suggested by several studies on *BRCA* analysis [7, 26]. In addition to fulfilling these conditions, all the pathogenic mutations in the 24 training samples were accurately identified without any false-positive alterations, including the large indels that were identified in two independent sequencing runs (Supplementary Table S1).

**Figure 4:**
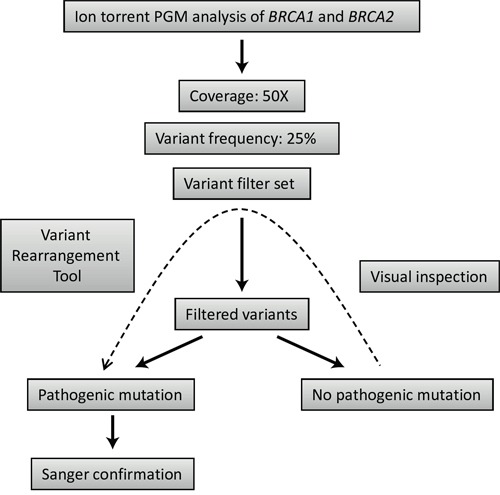
Strategy outline for mutation detection using Ion torrent PGM sequencing data

After the initial optimization, a subsequent group of 20 samples were selected for the validation of the diagnostic process on Illumina MiSeq and Ion Torrent PGM. Different NGS platforms have already been evaluated using the *BRCA1-2* genes as a model: some of these studies tested Ion Torrent PGM [26, 33], while others worked with Illumina HiSeq [7] or GS Junior from Roche 454 [34]. However, the direct comparison of these platforms using the same methodology in the context of *BRCA1* and *BRCA2* mutation detection performance is limited [27]. In our comparison of the two benchtop NGS platforms, Illumina MiSeq correctly identified all the pathogenic mutations in the 20 samples without the application of any of the variant filters. No false-positive mutations were identified. For Ion Torrent mutation detection, we used a set of variant filters developed to reduce sequencing error-derived false-positive results (Table 5). The main disadvantage of Ion Torrent semiconductor sequencing relative to Illumina chemistry is associated with the inaccuracy of length determination in homopolymers [3]. These errors tend to increase in genomic regions where the occurrence of true polymorphisms is also higher [35] and, thus, it is analytically challenging to reduce these errors without compromising detection sensitivity [36]. In our case, the application of the variant filter set developed within the frames of NGSeXplorer led to a drastic reduction of false-positive results illustrated by the specificity of 97.3%. Though, in one of the 20 samples a true pathogenic mutation was also discarded by one of the above mentioned filters resulting in an experimental sensitivity of 95.0%. Despite the fact that this false-negative case can be traced back to special reasons, missing even a single true-positive variant is unacceptable in a diagnostic setting. Visual inspection of the variant list before accepting the results of different filters could prevent the automatic rejection of true pathogenic mutations in the case of Ion Torrent sequencing (Figure 4).

A comprehensive diagnostic workflow for *BRCA1* and *BRCA2* must include the ability of specific and accurate detection of large genomic rearrangements in these genes. It has already been demonstrated that multiplex PCR reactions can be used to determine the copy number of the amplified targets by calculating their DQ in the case of multiplex amplicon quantification (MAQ) [37] or quantitative multiplex PCR of short fluorescent fragments (QMPSF) [38]. A precondition for CNV detection using the multiplex PCR/NGS method is that the normalized read counts are stable over different samples [39]. For this reason, homogeneously prepared, high-quality DNA must be used with similar amplification kinetics [26]. Our multiplex PCR/NGS sequencing pipeline provides promising results regarding LGR detection since the CNVs present were correctly identified (Figure 3).

In the recent years, the ability to detect *BRCA* mutations in tumor samples of ovarian cancer patients has become increasingly important [40]. For these reasons, we tested the performance of our method on DNA samples isolated from FFPE tissue blocks of 10 ovarian cancer patients. Despite the fact that based on the results of other studies [15, 17, 29] the frequency of somatic *BRCA* mutations in ovarian cancer is relatively low (5-8%), their detection is important since patients with somatic *BRCA* pathogenic variants may benefit from treatment with PARPi, similarly to patients with germline *BRCA* mutations [41]. In addition to FFPE DNA, we also tested blood DNA since we share the suggestion made by others [28] that FFPE analysis should not be used as a substitute for comprehensive germline *BRCA* analysis. From the four pathogenic *BRCA* mutations identified in the FFPE samples, 3 were confirmed to be present in the germline as well. Consequently, one out of the ten analyzed tumor samples contained a somatic mutation not reported before. This highlights the importance of *BRCA* mutation detection from FFPE tumor samples [42]. The frequency of the identified somatic mutation was higher than 80% suggesting the loss of the second non-mutated allele (loss of heterozygosity (LOH)) in the tumor (but not the normal tissue). However, the experiment that we carried out with mutant and non-mutant FFPE DNA mixtures demonstrated that our method is potentially able to detect mutations of low frequency (Supplementary Table S3).

To our knowledge, this is the first time the performance of an integrative NGS-based approach developed to perform comprehensive genetic testing of the *BRCA* genes has been evaluated on both germline and FFPE samples, simultaneously targeting a large spectrum of genetic alterations such as SNPs, indels, and LGRs. For germline mutations, the method was validated on the two most popular benchtop NGS platforms: Illumina MiSeq and Ion Torrent PGM. Our custom-designed NGS workflow for the genetic testing of germline *BRCA* mutations in combination with Illumina MiSeq sequencing meets the sensitivity and specificity requirements for the genetic diagnosis of HBOCS.

## MATERIALS AND METHODS

### Patients and DNA

DNA samples from cell lines with known deleterious variants in *BRCA1* (n=18; Table 1) or *BRCA2* (n=6; Table 1) were purchased from the Coriell Mutant Cell Repository (Camden, NJ). These reference samples contained both pathogenic and non-pathogenic variants. We also obtained 22 blood samples (Table 2) from patients previously diagnosed with *BRCA* mutations validated by Sanger sequencing (Kecskemét Hospital, HU). For FFPE testing, we received DNA samples from the Department of Pathology, University of Szeged (HU): ten ovarian tumor samples with matching non-tumorous FFPE or lymphocyte DNA. All patient samples were collected with appropriate consents approved by the regulatory and ethical authorities. Genomic DNA was isolated using PureLink™ Genomic DNA Mini Kit (Invitrogen, Carlsbad, CA-USA) for the blood samples and High Pure FFPET DNA Isolation Kit (Roche, IN, USA) for the FFPE samples.

### Multiplex PCR-based target amplification

The target region consisted of all coding exons of *BRCA1* (NM_007294.2) and *BRCA2* (NM_000059.3) as well as of the intronic sequences adjacent to each exon boundary. The adjacent intron length ranged between 10 and 50 bp, except for the 3′ end of *BRCA1* exon 9 and *BRCA2* exon 9, 14, and 17, where it was shorter due to primer design parameter constrains. Although mutations further into intronic sequences have been reported, their frequency appears to be low, and their significance is often unclear [43]. Overall, target regions are expected to encompass the majority of pathogenic sequence changes in *BRCA1* and *BRCA2* including all intronic splice site mutations.

Primer design was performed using the freely available software Primer3 [44]. All primers were checked for primer-dimer interactions, both for self-dimers and cross-dimers, to prevent template-independent primer extension. The resulting primers were ordered from Integrated DNA Technologies (Coralville, Iowa, USA) and tested in simplex PCR reactions on 20 ng of genomic DNA using 10 μM per primer; the other parameters were the same as those of the multiplex PCR described below. The multiplex PCR-based strategy considerably reduced the number of amplification reactions: four multiplex reactions for *BRCA1* and five multiplexes for *BRCA2* resulting in altogether 80 PCR fragments sized between 186 and 812 base pairs. Multiplex PCR reactions for *BRCA1* were performed in 35 μl volumes containing 1 × PCR buffer, 2.0 mM MgCl_2_, 2.5 mM dNTPs, 1.5 unit of Taq DNA polymerase (Thermo Scientific, Waltham, MA USA), and 20-50 ng of template DNA. Thermal cycler conditions were: 95°C for 2 min; 35 cycles of 95°C for 20 s; 63.5°C for 30 s; 72°C for 50 s; and, finally, 2 min at 72°C. *BRCA2* multiplexes were carried out in 25 μl volumes using 0.7 units Phusion High Fidelity Polymerase with 5xHF buffer and 10 mM dNTPs (Thermo Scientific). Thermal cycler conditions were: 98°C for 2 min; 35 cycles of 98°C for 20 s; 63.5°C for 30 s; 72°C for 50 s; and, finally, 2 min at 72°C. In all multiplex reactions, primer concentrations were optimized and varied between 0.05 pmol/μl and 0.2 pmol/μl final concentrations. PCR reactions were run on a 4% agarose gel for amplification quality control.

### Library construction and sequencing

After successful amplification of all *BRCA* fragments, multiplex PCR products belonging to the same sample were pooled together (40% - *BRCA1* and 60% - *BRCA2*) and purified with a PCR cleanup kit (Geneaid, Taiwan). The large size-range of amplicons (200-800 bp) in the multiplex PCR reactions requires further fragmentation to overcome the limitation of sequencing length for the Illumina and IonTorrent NGS platforms. We decided to use restriction enzyme-based fragmentation, a highly efficient and reproducible way of DNA fragmentation, which also makes the upstream step of adaptor ligation easier. We searched among enzymes with 4-nucleotide recognition sites as, theoretically, these provide an ideal fragmentation pattern of 250 bp long DNA. We carried out *in silico* fragmentation of the target genes with different 4-mer recognition enzymes that generate sticky ends using Clone Manager 9 (Scientific & Educational Software, Denver, CO, USA). The tested enzymes were ranked based on the number and size of fragments generated and the compatibility of sticky ends generated after digestion. Finally, three enzymes fulfilled our criteria: MseI (NEB, Ipswich, MA), Csp6I, and FspBI (Thermo Scientific). These restriction enzymes have an optimal cleavage site distribution along the target genes to generate DNA fragments between the size ranges of 50 and 300 base pairs (Figure 2). It was important to use more than one enzyme to ensure the presence of overlapping fragments similar to a random shearing with sonication. Most importantly, in case an enzyme site is inactivated by a point mutation the coverage of the respective region is ensured by the two neighboring enzyme sites. The possibility of two consecutive cleavage sites being inactivated was ignored. In most of the cases, additional enzyme sites were generated near the 5′ end of the primers to ensure the above mentioned optimal fragmentation conditions.

Restriction enzyme digestions were performed according to the manufacturer's instructions and were purified using the Small DNA Fragments extraction Kit (Geneaid, Taiwan). Custom barcoded adaptor sequences specific for Illumina or Ion PGM were ligated to the fragmented samples using T4 DNA Ligase (Thermo Scientific). Nick translation was carried out with DNA Polymerase I (Thermo Scientific). Generated DNA fragment libraries were checked for adapter dimers and size range (~170-400 bp) using gel electrophoresis on a 2% agarose gel. Samples were re-isolated from the gel with a DNA fragment extraction kit (Viogene, Taiwan). Fragment library quantification was carried out using the q-PCR-based quantification method (Kapa Biosystems) on LightCycler480 qPCR (Roche, Indianapolis, IN).

Illumina sequencing was carried out on the Illumina MiSeq system with Standard flow cell v2, following the manufacturer's instructions. For IonTorrent PGM sequencing, we used the Ion PGM™ Template OT2 200 Kit together with the Ion OneTouch™ 2 System (Thermo Fisher Scientific, Waltham, MA, USA). The generated ion sphere particles (ISP) were enriched using the ES module and were sequenced with an Ion PGM in a 200-bp configuration on 316 chip (Thermo Fisher Scientific, Waltham, MA, USA).

### Bioinformatic analyses

In addition to commercially available softwares, sequencing data were analyzed using the in-house developed NGSeXplorer bioinformatics software. A trial version of the software will be available at request from the authors.

The mapping algorithm of NGSeXplorer is composed of two distinct steps. In the first step, the software identifies alignment start positions using Burrows–Wheeler Transformation (BWT) [45]. The start position is defined as a 20-nucleotide-long key sequence from both ends of the reads (the first and last 4 nucleotides of each read is excluded from the search process). This key sequence is then tested against the reference in forward and reverse directions using the preset error threshold values. In this case, the default value is 2, which means that the alignment is acceptable only if the maximum difference between the key and the reference sequence does not exceed 2. In the second step, the software performs an accurate local alignment from the previously identified starting positions using the Smith-Waterman (SW) algorithm [46]. In our case, the scoring matrix of the SW algorithm prefers mismatches over indels as follows: match 10, mismatch -8, indel -9. There are no open gap or extended gap penalties in the scoring matrix. Starting positions of reads that are in close proximity to each other (<5 nucleotides) are combined together, and a mapping process is carried out with them. The first possible mapping result is picked out, and a linear run time rearrangement algorithm is applied on it to optimize for gaps and mismatches. Each rearrangement is scored based on the number of matches/mismatches and the number and length of indels. Our default scoring matrix is based on resequencing known samples and empirically determining the values to reach the maximum correctly mapped reads. The scoring matrix is: 100 for match, 30 for mismatch, 10 for deletion, and 1 for insertion; indels longer than 5 counted as match. In this way, the software can identify the best scored version of a read for the final alignment procedure. Since some primer positions occur within exons, alignments beginning at primer sites with sequences partially or exactly matching these primer sequences were soft-clipped. In other cases, primer sequences could hide real mutations during the variant calling process.

NGSeXplorer permits ready access to sample Binary alignment /Map format (BAM) and Variant Call Format (VCF) files. The VCF files are then further processed resulting in a preliminary classification of the observed variants: molecular classification (e.g. synonymous, missense, nonsense, frameshift) and clinical relevance based on locus-specific databases (BIC, UMD, Ensembl.org). Variant assessment is then performed manually by the operator by rechecking the quality metrics of the individual variants and its concordance with the IGV data. If the variant identification is accurate, the variants are loaded into a traceable database where the following fields are automatically filled by the NGSeXplorer software interface: Gene, Exon, Nucleotide, Change, dbSNP rs#, HGVS designation, and VAF (Variant Allele Frequency). All called variants were identified according to the Human Genome Variation Society (HGVS) nomenclature (http://www.hgvs.org/mutnomen) [47]. For variant calling from Ion Torrent sequencing data, we developed specific tests and a filter set with the purpose of reducing the high frequency of homopolymer sequencing errors specific to this platform:

#### Strand bias

The test filters out those variants that were detected only from one sequencing direction or whose frequency on the forward and reverse strands showed great difference (greater than 35%).

#### Depth test

Due to the enzymatic fragmentation of PCR fragments, sequencing of a nucleotide can start from several well-defined positions. The number of wild-type and mutant sequences arising from different start positions (different depths) is subjected to Mann–Whitney U test [48]. The test filters out those variants in which the pattern of depth distribution between wild-type and mutant reads shows a significant difference.

To further correct Ion Torrent homopolymer sequencing errors, we created a database from the sequencing data of 20 DNA samples that were considered wild-type for the *BRCA1* and *BRCA2* genes. The database contains the percentage values of insertion and deletion events for every base position, representing the basic error frequency of the platform for *BRCA1* and *BRCA2* sequencing. Those positions where the indel frequency is lower than 10% (reliable error) are considered low-error-frequency positions, and indel frequencies of newly sequenced samples measured for these positions are reliable and considered true variants if their frequency reaches or exceeds the indel detection limit of 25%. In high-error-frequency positions, where the frequency of indel events is higher than 10%, the following filters have to be applied to distinguish between sequencing errors and true indels:

#### Insertion overrun

This filter presumes that if a true single nucleotide insertion takes place in a homopolymeric region of a sample, this insertion frequency will be higher than the value measured in the database for the same insertion. Moreover, in the above mentioned case the frequency of two nucleotide insertions will also be significantly higher than in the database.

#### Deletion displacement

If a true single nucleotide deletion event takes place in a homopolymer region, it will result in a shift of the ratio of plus and minus 1 nucleotide-long indels relative to the ratio measured in the database for the same position; thus, when the ratio of deletions increases and the ratio of insertions decreases in the same sequence position it refers to a true deletion event.

#### Homopolymer (HP) percentage

Based on our observations, the ratio of an indel measured in a homopolymer position often requires correction due to the high frequency of sequencing errors in these regions. The correction we apply here is the difference between the frequencies of the opposite event in the database and in one particular sample, called **HP-opposite**. The frequency of an insertion or deletion corrected with this value is called **HP percentage**. The filter uses a well-defined decision-algorithm for the correction process (Supplementary Figure S2 and Supplementary Table S4).

#### Variant rearrangement tool

This experimental tool finds all non-homopolymer, larger than one nucleotide indels with low allele frequency and realigns all sequences near the found indels to maximize the possible length of insertions and deletions.

The overall mutation detection workflow using the above mentioned filters and tools is summarized in Figure 4.

### Large genomic rearrangements analysis

We evaluated the capacity of our multiplex PCR/NGS sequencing method to detect large genomic rearrangements (LGRs) using coverage data from Illumina MiSeq sequencing. Coverage data of two test samples (Table 2) were compared after normalization to the average of three reference samples with normal copy number. The normalization calculation and the comparison procedure of count data were performed separately for each of the four *BRCA1* multiplex PCRs as described previously [39]. Briefly, to determine the dosage quotient (DQ) of an amplicon in a given individual, the total absolute read count per specific multiplex was determined as the sum of the read counts for all amplicons of that specific multiplex. Per individual, the relative read count was determined for every amplicon as the ratio of the read count for that amplicon over the total absolute read count of the specific multiplex to which the amplicon belongs. The ratio of the relative read count of an amplicon of a test individual over the average relative read counts in the reference individuals results in the DQ for that amplicon in that individual. In short: DQ^i^_test_ = (RC^i^/RC^all^)_test_/(RC^i^/RC^all^)_ref_ with “RC”= read count; “i” = amplicon i; “all” = all amplicons; “test” = test individual; and “ref” = reference individual. The copy number of the samples used in these calculations was initially determined using the MLPA BRCA1 kit (MRC Holland, Amsterdam) according to the manufacturer's instructions.

### FFPE sample analysis

We amplified the *BRCA1* and *BRCA2* genes from the ten FFPE tumor samples and matching normal tissue/lymphocytes complementing the multiplex amplification with singleplex PCR reactions where it was necessary. We generated a fragment library and sequenced the samples on an Illumina MiSeq platform. For mutation detection, we used the NGSeXplorer with settings adapted for somatic variant calling: minimum coverage value of 100x and variant frequency threshold of 10%. We used mutant and non-mutant FFPE DNA mixtures in a serial dilution to demonstrate sensitivity limitations: one FFPE tumor sample with known BRCA pathogenic mutation (*BRCA1:* c.5263_5264insC) was mixed with non-mutant FFPE tumor DNA to make 1/2, 1/4, 1/8, and 1/16 admixtures.

## SUPPLEMENTARY TABLES AND FIGURES




