# Supplementary material for: Simultaneous detection of BRCA mutations and large genomic rearrangements in germline DNA and FFPE tumor samples
Source: Oncotarget. 2016 Aug 12;7(38):61845–59. doi: 10.18632/oncotarget.11259 (PMC5308695; doi:10.18632/oncotarget.11259)
Supplement: Supplementary file 1 [file oncotarget-07-61845-s001.pdf]

Simultaneous detection of *BRCA* mutations and large genomic rearrangements in germline DNA and FFPE tumor samples

SUPPLEMENTARY TABLES AND FIGURES

Supplementary Table S1: Reproducibility of large InDel detection

	c.3481_3491delGAAGATACTAG		c.1175_1214del40	
**Sequencing run**	NGS1	NGS2	NGS1	NGS2
**Mapped reads**	127,591	140,112	165,866	153,689
**Average coverage**	105.1x	160.5x	196.9x	485.2x
**Coverage at position**	303x	435x	334x	660x
**VAF**	36.30%	35.40%	53.89%	47.42%

Supplementary Table S2: Variant allele frequencies of tumour and matched normal DNA pairs resulting from Illumina MiSeq NGS sequencing

See Supplementary File 1

Supplementary Table S3: Detection of variant allele frequencies in serial mixture of mutant and non-mutant FFPE DNA samples

Variant	Dilution	Expected variant allele proportion*	Observed variant allele proportion	Coverage
**BRCA1: c.5382insC**	1/2	38.55%	30%	2552
	1/4	19.27%	10.78%	1776
	1/8	9.63%	3.92%**	3492
	1/16	4.81%	2.70%**	2552

* Expected frequency was based on results from the first sequencing run where the samples were undiluted
**Detected after visual inspection of filtered variants

Supplementary Table S4: Case examples for the HP percentage variant filter decision algorithm

Case	Variant	Sample	Data-base	DB%OE	DB%	HP Opposite (DB%OE-S%OE)	HP% (S%+HP Opposite)	HP% - DB%	(HP% - DB%) / DB%*100	Filter outcome
	1	28%	10%							
**a.**	0	73%	85%	3%<5%						True variant
	-1	2%	**3%**							
	1	35%	9%							
**b.**	0	65%	85%	8%>5	9%<10	3%	38%	29%>25%		True variant
	-1	5%	**8%**							
	1	30%	9%							
**c.**	0	65%	85%	8%>5	9%<10	-4%	26%	17%<25%		False variant
	-1	12%	**8%**							
	1	25%	20%							
**d.**	0	73%	85%	10%>5	20%<10	3%	28%		40%<25%	True variant
	-1	7%	**10%**							
	1	18%	20%							
**e.**	0	73%	85%	10%>5	20%<10	13%	21%		5%<25%	False variant
	-1	7%	**10%**							

+1 is one nucleotide insertion, 0 is Wt, -1 is one nucleotide deletion (Opposite Event – OE). For other abbreviations see Supp. Figure S1.

HP percentage filter here is tested against the insertion events of the sample (black frame).

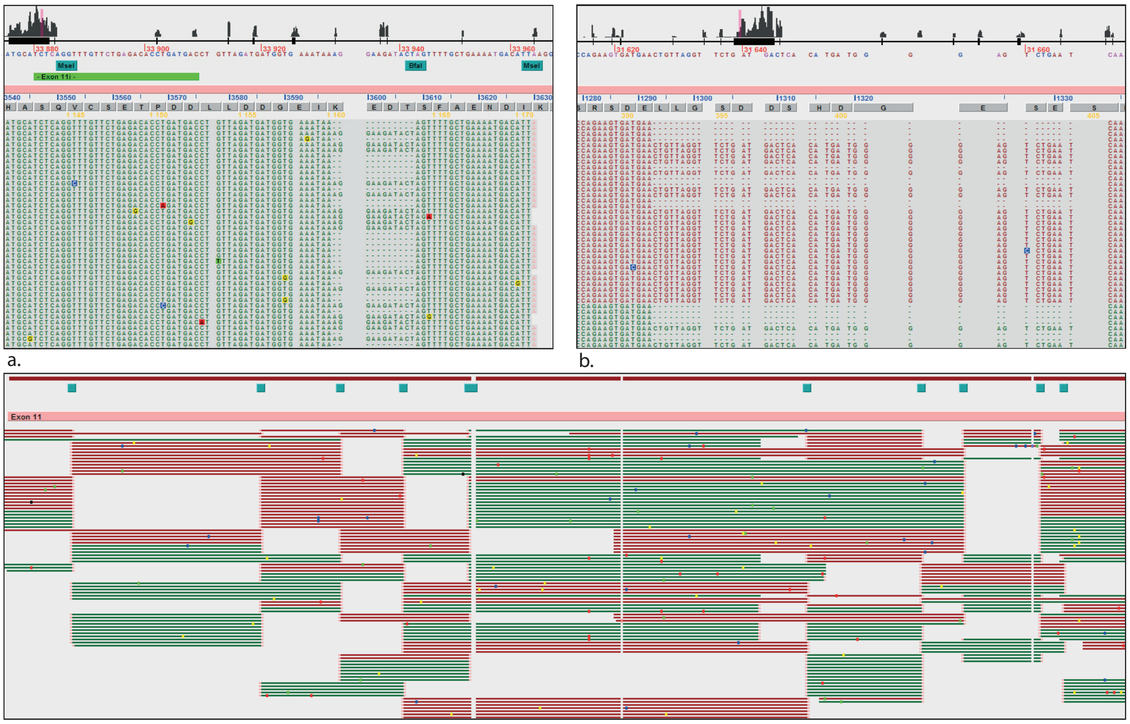

a. b. c.

Supplementary Figure S1: Graphic report of mapped reads generated by the NGSeXplorer. a. *BRCA1* c.3481_3491delGAAGATACTAG variant from sample GM14096. Green sequences represent the forward reads. Letters with coloured backgrounds mark the mismatches in the reads. The thick green line shows the position of forward primer used in the amplification of target sequences. **b.** *BRCA1* c.1175-1214del40 variant from sample NA14094. Red sequences represent the reverse reads. **c.** An overview of the coverage generated by overlapping restriction fragments at exon 11 of *BRCA2* in sample NA13712 in NGSeXplorer. Green lines represent forward, red lines represent reverse reads. Each line is a group of the reads with the same sequence. Coloured squares are the mismatches and insertion present in the reads.

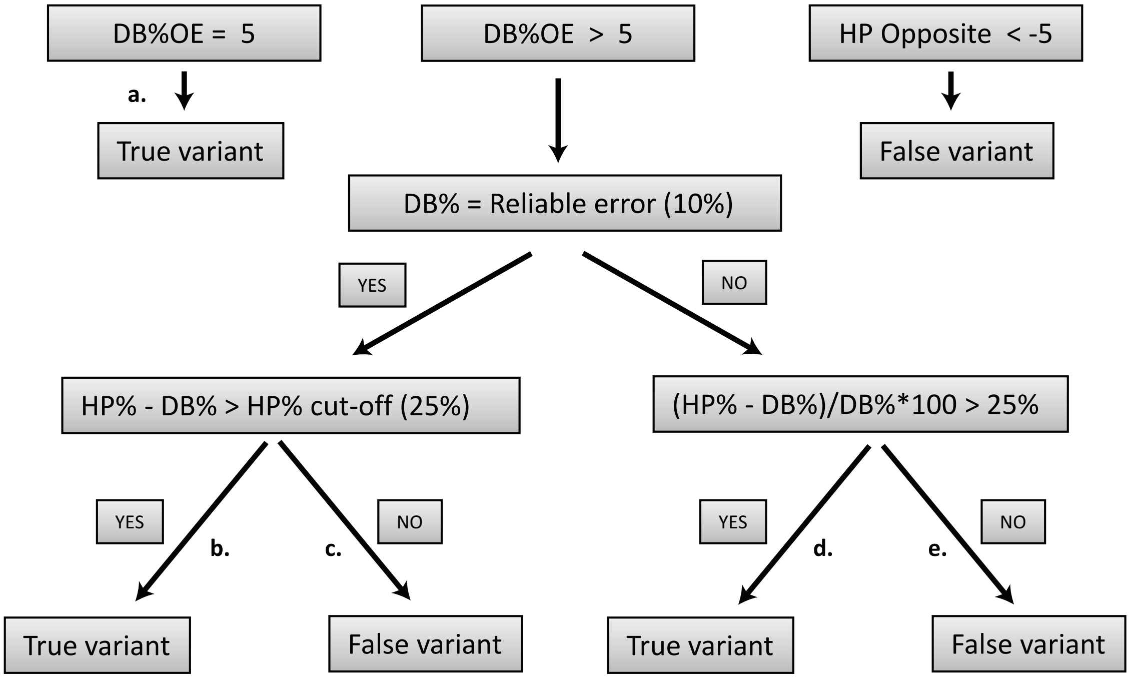

Supplementary Figure S2: Decision algorithm for the HP percentage (HP%) variant filter. HP Opposite=DB%OE-S%OE; HP%=S%+HP Opposite. Abbreviations: DB%: percentage of the variant in the database, DB%OE: percentage of the opposite event in the database, S%: percentage of the variant in the sample, S%OE: percentage of the opposite event in the sample.
