# Supplementary material for: Simultaneous detection of BRCA mutations and large genomic rearrangements in germline DNA and FFPE tumor samples
Source: Oncotarget. 2016 Aug 12;7(38):61845–59. doi: 10.18632/oncotarget.11259 (PMC5308695; doi:10.18632/oncotarget.11259)
Supplement: Supplementary file 2 [file oncotarget-07-61845-s002.docx]

**Supp. Table S2.** Variant allele frequencies of tumour and matched normal DNA pairs resulting from Illumina MiSeq NGS sequencing.

| **Gene** | **Variant** | | | **Case 1** | | **Case 2** | | **Case 3** | | **Case 4** | | **Case 5** | | **Case 6** | | **Case 7** | | **Case 8** | | **Case 9** | | **Case 10** | |
| --- | --- | --- | --- | --- | --- | --- | --- | --- | --- | --- | --- | --- | --- | --- | --- | --- | --- | --- | --- | --- | --- | --- | --- |
|  | **HGVS cDNA** | **HGVS Protein** | **dbSNP** | Blood | Tumor | Bood | Tumor | Blood | Tumor | Blood | Tumor | Blood | Tumor | Blood | Tumor | Normal | Tumor | Normal | Tumor | Blood | Tumor | Blood | Tumor |
| BRCA1 | c.-19-115T>C | p. IVS1-115 T>C | rs3765640 |  |  |  |  |  |  |  |  | **55%** | **66%** | **53%** | **61%** | **65%** | **91%** | **41%** | **23%** |  |  |  |  |
| BRCA1 | c.441+63_441+74delCTTTTTTTTTTT | p. IVS7+36 delCTTTTCTTTTTTTT | rs373413425 |  |  | **46%** | **53%** |  |  |  |  | **38%** | **61%** | **36%** | **49%** | **59%** | **87%** | **45%** | **26%** |  |  |  |  |
| BRCA1 | c.442-34C>T | p. IVS7-34 C>T | rs799923 | **42%** | **16%** |  |  | **47%** | **38%** |  |  |  |  |  |  |  |  |  |  |  |  |  |  |
| BRCA1 | c.547+146A>T | p. IVS8+146 A>T | rs8176140 |  |  | **50%** | **68%** |  |  |  |  | **56%** | **72%** |  |  |  |  | **45%** | **36%** |  |  |  |  |
| BRCA1 | c.548-68_548-68delT | p. IVS8-58 delT | rs273902772 |  |  |  |  |  |  |  |  | **62%** | **73%** | **50%** | **66%** |  |  | **13%** | **38%** |  |  |  |  |
| **BRCA1** | **c.628C>T** | **p. Q210*** | **-** |  |  |  |  |  |  |  |  |  |  |  |  | **0%** | **87%** |  |  |  |  |  |  |
| BRCA1 | c.2077G>A | p. D693N | rs4986850 |  |  | **23%** | **57%** |  |  |  |  |  |  |  |  |  |  |  |  |  |  |  |  |
| BRCA1 | c.2082C>T | p. S694 | rs1799949 |  |  | **36%** | **57%** |  |  |  |  | **46%** | **16%** | **49%** | **57%** | **51%** | **93%** | **47%** | **13%** |  |  |  |  |
| BRCA1 | c.2311T>C | p. L771 | rs16940 |  |  | **52%** | **61%** |  |  |  |  | **51%** | **30%** | **46%** | **60%** | **56%** | **92%** | **54%** | **12%** |  |  |  |  |
| BRCA1 | c.2591C>T | p. S864L | rs80357003 |  |  |  | **17%** |  |  |  |  |  |  |  |  | **45%** | **91%** |  |  |  |  |  |  |
| BRCA1 | c.2612C>T | p. P871L | rs799917 |  |  | **48%** | **66%** |  |  |  |  | **49%** | **75%** | **43%** | **58%** |  |  | **48%** | **30%** |  |  |  |  |
| BRCA1 | c.2635G>A | p. E879K | rs80357251 |  |  |  |  |  |  |  |  | **0%** | **70%** |  |  |  |  |  |  |  |  |  |  |
| BRCA1 | c.3113A>G | p. E1038G | rs16941 |  |  | **53%** | **31%** |  |  |  |  | **49%** | **29%** | **49%** | **58%** | **46%** | **89%** | **54%** | **25%** |  |  |  |  |
| BRCA1 | c.3548A>G | p. K1183R | rs16942 |  |  | **49%** | **56%** |  |  |  |  | **49%** | **26%** | **50%** | **60%** | **49%** | **89%** | **51%** | **35%** |  |  |  |  |
| BRCA1 | c.4097-141A>C | p. IVS11-141 A>C | rs799916 |  |  | **47%** | **34%** |  |  |  |  | **47%** | **24%** | **50%** | **58%** | **43%** | **94%** | **51%** | **13%** |  |  |  |  |
| BRCA1 | c.4308T>C | p. S1436 | rs1060915 |  |  | **53%** | **60%** |  |  |  |  | **45%** | **90%** | **36%** | **49%** | **52%** | **92%** | **50%** | **25%** |  |  |  |  |
| BRCA1 | c.4357+117G>A | p. IVS13+117 G>A | rs3737559 |  |  |  |  |  |  |  |  |  |  |  |  | **49%** | **91%** |  |  |  |  |  |  |
| BRCA1 | c.4837A>G | p. S1613G | rs1799966 |  |  | **54%** | **60%** |  |  |  |  | **45%** | **75%** | **47%** | **58%** | **48%** | **92%** | **50%** | **24%** |  |  |  |  |
| BRCA1 | c.4986+222A>G | p. IVS16+222 A>G | rs3092987 |  |  |  |  |  |  |  |  | **64%** | **53%** | **41%** | **68%** | **74%** | **92%** | **56%** | **48%** |  |  |  |  |
| BRCA1 | c.5152+66G>A | p. IVS18+66 G>A | rs3092994 |  |  |  |  |  |  |  |  | **37%** | **70%** | **46%** | **58%** | **47%** | **90%** | **47%** | **24%** |  |  |  |  |
| **BRCA1** | **c.5251C>T** | **p. R1751*** | **rs80357123** |  |  |  |  |  |  |  |  |  |  |  |  |  |  |  |  |  |  | **44%** | **92%** |
| **BRCA1** | **c.5263_5264insC** | **p.Ser1755?fs** | **rs80357906** |  |  |  |  |  |  |  |  |  |  |  |  |  |  | **50%** | **70%** | **49%** | **63%** |  |  |
| BRCA2 | c.-26G>A | - | rs1799943 |  |  | **87%** | **99%** |  |  |  |  |  |  |  |  |  |  | **49%** | **48%** |  |  |  |  |
| BRCA2 | c.68-7T>A | p. IVS2-7 T>A | rs81002830 |  |  |  |  |  |  |  |  |  |  |  |  |  |  | **60%** | **37%** |  |  |  |  |
| BRCA2 | c.425+67A>C | p. IVS4+67 A>C | rs11571610 |  |  |  |  |  |  |  |  |  |  | **67%** | **66%** |  |  |  |  |  |  |  |  |
| BRCA2 | c.426-89T>C | p. IVS4-89 T>C | rs3783265 |  |  |  |  |  |  |  |  |  |  | **48%** | **48%** |  |  |  |  |  |  |  |  |
| BRCA2 | c.865A>C | p. N289H | rs766173 |  |  |  |  |  |  |  |  |  |  | **40%** | **41%** |  |  |  |  |  |  |  |  |
| BRCA2 | c.1114C>A | H372N | rs144848 |  |  |  |  | **47%** | **73%** | **46%** | **83%** | **53%** | **77%** |  |  |  |  |  |  |  |  |  |  |
| BRCA2 | c.1365A>G | p. S455 | rs1801439 |  |  |  |  |  |  |  |  |  |  | **35%** | **40%** |  |  |  |  |  |  |  |  |
| BRCA2 | c.1910-74T>C | p. IVS10-74 T>C | rs2320236 |  |  |  |  |  |  |  |  |  |  |  |  |  |  | **60%** | **100%** |  |  |  |  |
| BRCA2 | c.1910-51G>T | p. IVS10-51 G>T | rs11571651 |  |  |  |  |  |  |  |  |  |  | **54%** | **40%** |  |  |  |  |  |  |  |  |
| BRCA2 | c.2229T>C | p. H743 | rs1801499 |  |  |  |  |  |  |  |  |  |  | **50%** | **42%** |  |  |  |  |  |  |  |  |
| BRCA2 | c.2971A>G | p. N991D | rs1799944 |  |  |  |  |  |  |  |  |  |  | **43%** | **43%** |  |  |  |  |  |  |  |  |
| BRCA2 | c.3396A>G | p. K1132 | rs1801406 |  |  | **96%** | **100%** |  |  |  |  |  |  |  |  |  |  | **14%** | **65%** |  |  |  |  |
| BRCA2 | c.3807T>C | p. V1269 | rs543304 | **94%** | **100%** |  |  | **47%** | **37%** |  |  | **47%** | **14%** |  |  | **53%** | **52%** |  |  |  |  | **99%** | **99%** |
| BRCA2 | c.7806-14T>C | p. IVS16-14 T>C | rs9534262 |  |  |  |  |  |  | **56%** | **87%** |  |  | **96%** | **98%** | **33%** | **39%** | **51%** | **45%** | **48%** | **53%** |  |  |
| BRCA2 | c.9257-16T>C | p. IVS24-16 T>C | rs11571818 |  |  |  |  |  |  |  |  |  |  |  |  |  |  |  |  | **51%** | **50%** |  |  |

Pathogenic variants and mutant samples are highlighted and bold in red.
